# Cholera Precautions

**Published:** 1892-09-17

**Authors:** Andw. Clark, Wm. Henry Allchin

**Affiliations:** President of the Royal College of Physicians; Vicarius for Edward Liveing, M.D., Registrar. To Sir Hugh Owen, K.C.B.


### CHOLERA PRECAUTIONS.

IN response to a request, made by the President of the Local Government Board, a special sederunt of the Royal College of Physicians has prepared the following series of cholera instructions and regulations for the use of the medical officers of the Board, and for the guidance of the public :—

Royal College of Physicians, September 3rd, 1892.

SIR,—In complying with the request of the Local Government Board to furnish it with instructions for the management of health in view of the prevalence of diarrhœa and of cholera, the Royal College of Physicians desires to say that the instructions herewith submitted are not intended either to occupy the general ground of prevention so ably and admirably covered by the medical advisers of the Board, or to supersede the necessity of immediately summoning medical assistance to those stricken with disease. They are meant to be followed only when the assistance of a doctor cannot be procured, and when diarrhœa has not developed into cholera. The College proposes no instructions for the treatment of cholera. Every case of this disease requires separate consideration and management; no stereotyped plan of treatment would prove to be either wise or safe; and, usually before the choleraic nature of an attack could be established, medical assistance would have been procured.

The chief instructions to be followed for the prevention of diarrhœa and of cholera are herewith appended :—

1. As cholera is not in the ordinary sense of the term contagious, as it is rarely, if ever, communicated, like small-pox or scarlet fever, directly from person to person, as it is probable that those engaged in attendance upon patients suffering from this malady are not more liable than others to become attacked with it, and as it is certain that physical and moral depression favour the reception and development of the disease, apprehensions should be allayed, confidence encouraged, and that manner of living pursued which experience has proved to be conducive to the highest health.

2. The house should be clean, light, thoroughly dry, and well ventilated. Air shafts, traps, and drains should be in perfect working order. Dustbins should be frequently emptied, and no decaying matters of any kind should be permitted to remain in or near the house. Cisterns, reservoirs, casks, jars, and pipes used in the preserving, carrying, or transmitting of water should be frequently inspected and carefully cleansed. All connections of waste pipes with drains should be severed.

3. As water is one of the chief agents by which choleraic infection is conveyed, all water employed for personal or domestic use in the household should be scrupulously protected from contaminations of every kind; and if any doubts of its purity arise, the water should be boiled, filtered, and consumed within twenty-four hours. Boiled and filtered rain water is probably the best of all waters for use at this time.

4. The dietary should consist daily of three or four simple but nourishing and ample meals taken at regularly recurring times. The meals may consist of any sort of animal food, fresh and thoroughly cooked, of bread, potatoes, well-boiled green vegetables, if they agree, and of plain farinaceous puddings, or of simply cooked wholesome fruit.

Milk should be boiled before use.

Alcoholic beverages should be taken in great moderation, and only at the greater meals, such as at dinner and supper.

It is desirable to avoid soups, tinned or otherwise preserved provisions, raw or stale vegetables, unripe, overripe, or decaying fruits, pastry, cheese, nuts, hard or indigestible things of every kind, malt liquors turning "hard," ginger beer, strongly ascescent sparkling wines, coarse oatmeal gruel, messes between meals, and either long fasts or too frequent feeding.

5. All provisions should be procured fresh and fresh, but when some storage is unavoidable the most scrupulous care should be taken to protect them from contamination by impure air or water.

Cooking utensils should be scalded after use and kept carefully clean.

6. Avoid the use of strong aperients, and especially of strong saline aperients. If there is obstinate constipation, take at bedtime either a teaspoonful of Gregory's powder, or one or two teaspoonfuls of castor oil.

7. Avoid excess and irregularities of every kind, over fatigue, prolonged watchings, emotional excitements, undue mental strain, and all such things as irritate and exhaust the nervous system.

Especially avoid the frequent use of alcoholic or of any stimulants to cover recurring sensations of sinking, malaise, or depression.

8. Take moderate exercise, twice daily; follow early hours; and aim at leading a regular, an occupied, and a tranquil life.

CONCERNING THE MANAGEMENT OF LOOSENESS OF THE BOWELS.

9. If, notwithstanding this careful regulation of the manner of living, looseness of the bowels should set in, send immediately for medical assistance, since, without personal examination and direction, no case of this kind, arising in such circumstances, can be satisfactorily or even safely managed. But if medical assistance is not immediately available, follow the subjoined instructions until the doctor arrives.

10. Choose, if practicable, a bright, airy room, go at once to bed, keep quite warm, and if troubled with cramps or pains, apply hot applications to the entire stomach.

11. Take freshly prepared fluid or semi-fluid food in quantities of a large cupful at a time regularly every three hours. Such food may consist of boiled milk, thickened with rice flour, baked flour, or biscuit powder; of tea made with boiling milk infused about five minutes, and having toast, biscuits, or rusks soaked in it; of farinaceous puddings of the nursery sort; of any kind of gruel, except that made with coarse oatmeal; of meat jelly, of beef tea, or of mutton, chicken, or veal broth.

If pain persists with depression or faintness, take a tablespoonful of brandy or of whiskey in a small claret glassful of hot water after meals, twice, thrice, or four times in the course of the twenty-four hours; but not oftener than is absolutely required for relief.

12. If thirst becomes excessive, sip from time to time small quantities of iced water just sensibly acidulated with fresh juice of lemons or with aromatic sulphuric acid.

13. As soon as possible after looseness of the bowels has begun, take in capsules, or in hot milk, or in any other manner preferred, two teaspoonfuls of castor oil. If when the action of the oil may be fairly supposed to have ceased the looseness increases to a watery diarrhœa, let the hips be well raised and carefully inject into the bowels a quart or more of hot water containing two drachms of benzoate of soda or thirty grains of tannin. Furthermore, if there be much pain in the bowels, fifteen to thirty drops of laudanum may be added to the injection. The injection should be retained as long as it is comfortable to the patient, and it may be repeated once or twice daily during the continuance of the diarrhœa, and until medical assistance has been procured.

14. After the administration of the injection, if one has been found necessary, the following mixture should be taken at intervals of from three to four hours, according to the urgency of the symptoms :—

Mist. Cretæ Aromat. one ounce, Tinct. Camph. Comp. half a dram, Tinct. Chlorofor. Comp. twenty minims, Sp. Ammon. Arom. twenty minims, Cerii et Bismuthi Salicyl. five grains, Ess. Menthæ Pip. ten minims. Fiat dos. 1.

15. Should this mixture disagree, or in four-and-twenty hours fail to give relief, the mixture following should be substituted and taken every three or four hours :—

Acid. Sulph. Arom. fifteen minims, Tinct. Camph. Comp. half a dram, Tinct. Chlorofor. Comp. twenty minims, Tinct. Coto. twenty minims, Syrupi Aurant. Flor. one dram, Aq. Menthæ Pip. ad one ounce. Fiat dos. 1.

16. From the first appearance of looseness of the bowels the body should be washed with warm water night and morning, and quickly dried; soiled bed or other clothing should be immediately disinfected and destroyed.

A cheap and efficient disinfecting fluid is recommended by Dr. Thorne Thorne, and is thus prepared :—Dissolve half an ounce of corrosive sublimate and five grains of commercial aniline blue in three gallons of water, and add thereto one fluid ounce of hydrochloric acid. Preserve in earthenware jars or wooden tubs. I have, &c.,

(Signed) ANDW. CLARK, M.D., President of the Royal College of Physicians.
WM. HENRY ALLCHIN, M.B., Vicarius for Edward Liveing, M.D., Registrar.

To Sir Hugh Owen, K.C.B.

